# ortho2align: a sensitive approach for searching for orthologues of novel lncRNAs

**DOI:** 10.1186/s12859-022-04929-y

**Published:** 2022-09-19

**Authors:** Dmitry Evgenevich Mylarshchikov, Andrey Alexandrovich Mironov

**Affiliations:** 1 Faculty of Bioengineering and Bioinformatics, Lomonosov Moscow State University, Moscow, Russian Federation 119234; 2 Institute for Information Transmission Problems, Russian Academy of Sciences, Moscow, Russian Federation 127994

**Keywords:** lncRNAs, Evolution, Orthology, Software

## Abstract

**Background:**

Many novel long noncoding RNAs have been discovered in recent years due to advances in high-throughput sequencing experiments. Finding orthologues of these novel lncRNAs might facilitate clarification of their functional role in living organisms. However, lncRNAs exhibit low sequence conservation, so specific methods for enhancing the signal-to-noise ratio were developed. Nevertheless, current methods such as transcriptomes comparison approaches or searches for conserved secondary structures are not applicable to novel, previously unannotated lncRNAs by design.

**Results:**

We present ortho2align—a versatile sensitive synteny-based lncRNA orthologue search tool with statistical assessment of sequence conservation. This tool allows control of the specificity of the search process and optional annotation of found orthologues. ortho2align shows similar performance in terms of sensitivity and resource usage as the state-of-the-art method for aligning orthologous lncRNAs but also enables scientists to predict unannotated orthologous sequences for lncRNAs in question. Using ortho2align, we predicted orthologues of three distinct classes of novel human lncRNAs in six Vertebrata species to estimate their degree of conservation.

**Conclusions:**

Being designed for the discovery of unannotated orthologues of novel lncRNAs in distant species, ortho2align is a versatile tool applicable to any genomic regions, especially weakly conserved ones. A small amount of input files makes ortho2align easy to use in orthology studies as a single tool or in bundle with other steps that researchers will consider sensible. ortho2align is available as an Anaconda package with its source code hosted at https://github.com/dmitrymyl/ortho2align.

**Supplementary Information:**

The online version contains supplementary material available at 10.1186/s12859-022-04929-y.

## Background

Many new long noncoding RNAs have been discovered in human cell lines in recent years due to advances in high-throughput RNA sequencing experiments. For instance, novel chromatin-associated RNAs (X-RNAs) were discovered in a Red-C experiment [1], are enriched in active or repressed chromatin and may modulate chromatin structure; short tandem repeat-enriched RNAs (strRNAs) might interact with multiple copies of specific RNA-binding proteins [2]; and semi-extractable RNAs (seRNAs) are thought to comprise novel nuclear bodies [3]. However, some of them might come from transcriptional noise [4–6] or aberrations during transcript assembly. Conservation studies of novel lncRNAs might support their existence as functional genomic units and support their functional roles in cells as well as shed light on their mechanism of action [7]. But, lncRNAs exhibit low sequence conservation [8], so specific methods for enhancing the signal-to-noise ratio are needed. The first group of such methods leverages experimental evidence and compares transcriptomes of two or more species, which results in direct orthologues assignment between two transcriptomes but restricts discovery of unannotated orthologues. Methods in this group use two strategies to enhance signal-to-noise ratio: either assigning orthologues based on syntenic relations (PLAR [7], slncky [9], lncEvo [10]) or based on the best reciprocal hits (BRH) from pairwise sequence alignments (Evolinc [11]). While BRH strategy is exhaustive in terms of computational time and might result in a certain number of false matches, synteny-based approaches sensibly reduce search space and align only those transcripts that are encoded in corresponding syntenic regions, which leads to reduced computation time and fewer false matches. Still, the above mentioned methods that compare transcriptomes of two species are not applicable to novel lncRNAs discovered in single original experiments, as there will be no congruent experimental data from the target species, which is the case for X-RNAs, strRNAs and seRNAs. The second group searches for conserved secondary structures (RNAz [12], EvoFold [13]) since RNAs are folded into some secondary structures that might facilitate their functions. However, novel lncRNAs are usually not assigned with any covariational matrix and might not require any conserved secondary structure for carrying out their functions [14] so applying those methods to lncRNAs is not correct. To fill in the gap and aid the search of orthologues of novel lncRNAs discovered in single original experiments we developed ortho2align—a sensitive synteny-based approach with a statistical assessment of sequence conservation. We estimated the performance of the method on a published lncRNAs orthologues dataset, compared ortho2align with a baseline synteny-based approach and a state-of-the-art transcriptome comparison approach, and predicted orthologues for 3 distinct classes of novel lncRNAs.

## Approach and implementation

**Fig. 1:**
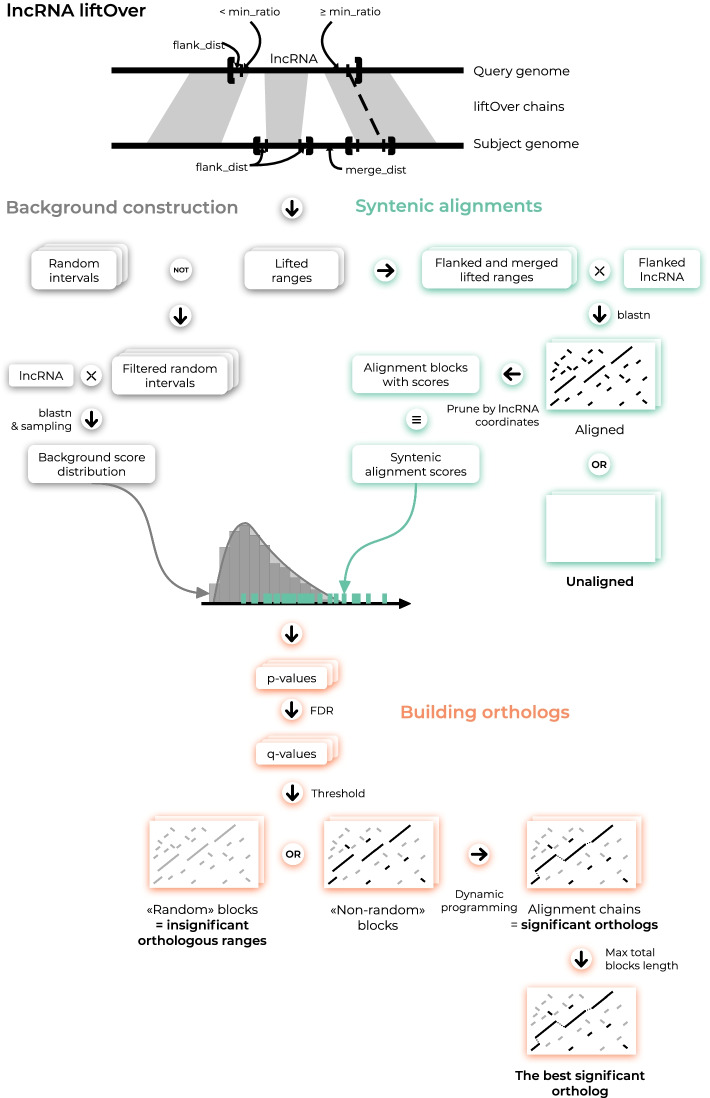
ortho2align algorithm for a single lncRNA

The formal problem is as follows: to find a single orthologue in a subject genome for every lncRNA in a given annotation of the query genome. The ortho2align pipeline breaks the solution into several steps, which are described below (see also Fig. 1).

### Building syntenies

First, lncRNA coordinates are lifted from the query species genome to the subject species genome with liftOver [15] with allowed duplications and minMatch = 0.05 by default. Next, syntenic regions are constructed from those lifted coordinates. Duplications are merged if they are closer than the specified distance. Constructed syntenies are flanked with a specified number of nucleotides.

### Getting alignments

Flanked lncRNAs are aligned to their syntenic regions with BLASTN [16] with loose parameters (word_size = 6 by default) which results in a set of high-scoring segment pairs (HSPs) for every syntenic region for every lncRNA pruned by lncRNA coordinates. lncRNAs that were successfully lifted but have zero HSPs are deemed as *unaligned* and their lifted coordinates in subject species are reported separately.

### Estimating background

To remove spurious HSPs a background distribution of raw HSP scores is constructed for every lncRNA by aligning its sequence to a sample of shuffled genomic ranges from the annotation of the subject genome. To ensure potential orthologues are not included in the set of background ranges, any background range is removed in case it intersects lifted coordinates of one or more lncRNAs.

### Filtering HSPs

For every lncRNA every HSP is assigned a p-value based on a right-sided statistical test in which the HSP score is so large only for random reasons based on the background distribution of raw HSP scores. P-values are adjusted with the Benjamini-Hochberg procedure [17]. Only HSPs with a q-value less than a specified α value are retained to control the false discovery rate at α ∙ 100% (5% by default). lncRNAs with no significant HSPs retained are deemed as *insignificant* orthologues and all their HSPs are reported separately.

### Building orthologues

Retained HSPs are linked via dynamic programming to form alignment chains, one per every syntenic region of every lncRNA. Those chains are deemed *significant* orthologues and reported with their significant HSPs. There might be multiple *significant* orthologues produced for any single query lncRNA.

### Selecting the best significant orthologue for every lncRNA

In case one wants to obtain one-to-one orthologues, only one orthologue for every lncRNA is selected based on the maximal sum of lengths of HSPs that comprise the orthologue in question. These one-to-one orthologues are named “*bestSignificant*” in the programme output.

### Annotating orthologues (optional)

Found orthologues can be annotated with a provided gene annotation of the subject genome by intersecting two gene sets in a strand-specific manner. Jaccard indexes and overlap coefficients of intersecting orthologues and subject species genes are also reported.

### Implementation details

Running BLASTN with loose parameters increases sensitivity, but applying it to the whole genome dramatically increases the working time and the number of false HSPs. Restricting the search space to syntenic regions overcomes these issues. Additionally, every lncRNA is aligned to a sample of shuffled genomic ranges of the subject genome to estimate how well it aligns to random places of the subject genome. The generated distribution of random HSP scores is used to assess whether syntenic HSPs represent truly conserved sequences or are not significantly different from random HSPs. The latter procedure enhances the specificity of the algorithm.

Besides the best significant orthologues, ortho2align reports all significant orthologues, insignificant orthologues, and lifted coordinates of unaligned lncRNAs to provide one with complete information on conservation status of as many lncRNAs as possible.

ortho2align is implemented in python 3 and requires only two non-python dependencies: standalone BLAST and liftOver programmes. Due to algorithm being atomic to a single lncRNA, ortho2align benefits much from parallelization on multiple cores. ortho2align is designed to leave a small RAM footprint by dumping intermediate files into disk so parallelization will not affect RAM usage much.

ortho2align requires only five input files: query species lncRNAs annotation, query species genome file, subject species genome file, subject species gene annotation for background ranges construction and optional annotation and a liftOver chain file (Additional file 1: Fig. S1). All these files are available from UCSC and other major annotation providers as is, so gathering them would not be a problem. Also, liftOver chain file can be generated for genome assemblies that are not stored at UCSC. As intermediate files are preserved, each step of the pipeline can be run separately (Additional file 1: Fig. S1), in case one wants to adjust algorithm parameters of downstream steps without rerunning upstream steps.

## Results

### Performance estimation

**Fig. 2:**
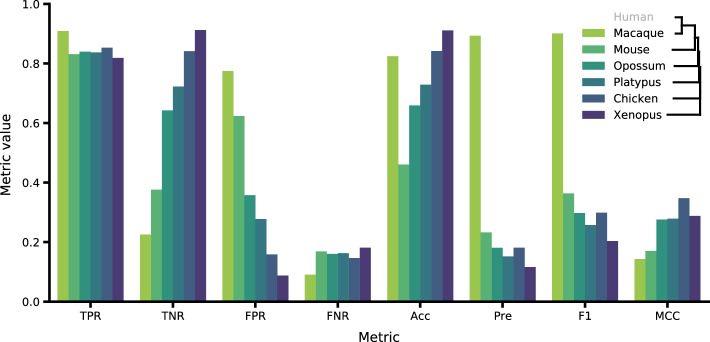
ortho2align performance metrics on the benchmarking dataset

To estimate the performance of the method we took a public dataset of lncRNA orthologues in various species [18]. We predicted orthologues of all 14,682 human lncRNAs in 6 species: rhesus macaque, mouse, opossum, platypus, chicken and *Xenopus tropicalis—*and compared the best significant predicted orthologues with true annotated ones. True positive rate (TPR) was over 80% in all species, even the distant ones, which means high sensitivity of ortho2align taking into account different evolutionary distances between human and subject species. False positive rate (FPR) dropped from 80 to 10% with increase in evolution distance between human and subject species whereas accuracy increased along the specified direction (see Fig. 2). Precision was low except for macaque probably due to high class imbalance: there are much fewer human lncRNAs conserved in distant species than non-conserved as labeled in the benchmarking dataset (see Additional file 1: Table S2).

**Fig. 3:**
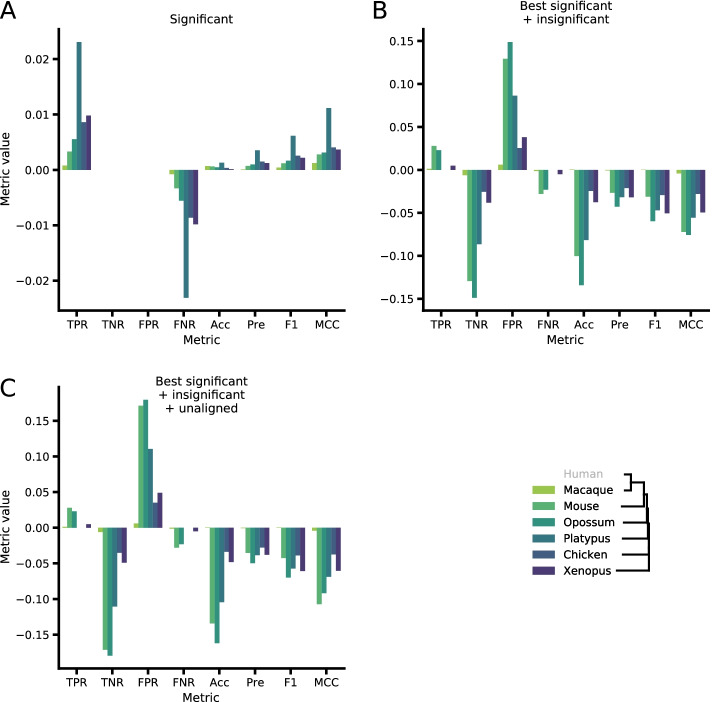
ortho2align performance metrics gain compared to the best significant orthologues when added other sets of predicted orthologues. **A** Performance metrics gain when considering all significant orthologues compared to considering the best significant orthologues alone. **B** Same as A, but comparing a combination of the best significant and insignificant orthologues to the best significant orthologues alone. **C** Same as B, but comparing a combination of the best significant, insignificant and unaligned orthologues to the best significant orthologues alone

Taking into account all significant orthologues resulted in a small gain of TPR (up to 2.3%) but no increase in false positive predictions, which suggests an incorrect resolution of paralogues either in ortho2align or in the benchmarking dataset (Fig. 3). Combining the best significant orthologues with the insignificant ones and also with unaligned lifted regions resulted in a small gain of TPR (up to 2.8%), but led to substantial increase in FPR (up to 15%). These statistics clearly state that HSPs filtering and orthologues selection steps do increase specificity and precision of the procedure.

### Comparison with similar tools

**Fig. 4:**
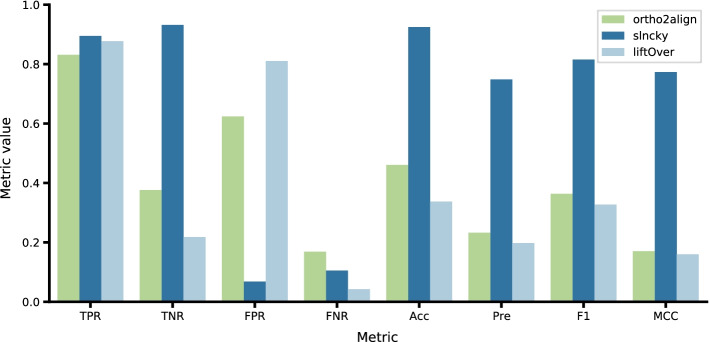
Performance metrics of ortho2align, slncky and liftOver on predicting mouse orthologues

To our knowledge, there are no specific tools solving the same problem: discovery of orthologues in subject species of previously unannotated lncRNAs of query species with no congruent experimental data in subject species. So to find out how our method compares to other methods in the field, we took two similar tools. The first one is slncky—a state-of-the art synteny-based method for lncRNAs orthologue assignment based on comparison of transcriptomes of two species. The second one is a naive liftOver with allowed duplications—a baseline approach of lifting coordinates between syntenic regions. Due to slncky requiring numerous annotation files we restricted our comparison to finding orthologues of human lncRNAs in mouse as numerous files required for that were already provided by slncky.

slncky outperformed other methods in all metrics except for false negative rate (FNR), where liftOver was the best. ortho2align showed the lowest TPR, though differences between methods are quite small, but the second lowest FPR (see Fig. 4). Despite not having outperforming metrics, ortho2align has two design advantages over the other tools. First, slncky searches for orthologues if the syntenic region contains one or more annotated lncRNAs, which results in a small FPR by design but also makes it impossible to detect unannotated orthologues in subject species. It is possible that several to many orthologues have not yet been observed in any experiment (like with X-RNAs, strRNAs and seRNAs), so predicting unannotated orthologues is valuable and high FPR of ortho2align might come from those *bona fide* unannotated orthologues, hence slncky fails to solve the problem in question while ortho2align does the job. Second, liftOver does not permit duplications by default, which might result in a significant loss of orthologues, or doesn’t provide a strategy to select one orthologue out of many for a single lncRNA in case duplications are allowed. ortho2align does provide this strategy, but also saves information about potential paralogues. Finally, one should take into account that there is no gold standard for this task and TNs and FPs are not well defined. So, ortho2align shows a comparable performance to similar tools in the field, especially sensitivity, or TPR, while being the only tool that solves the task in question: discovery of orthologues of novel lncRNAs of query species with no available lncRNAs annotation and congruent experimental data for subject species.

### Resources usage

All ortho2align, slncky and liftOver launches were done on a single node of two Intel Xeon X5670 processors. ortho2align and slncky were parallelized on 20 cores.

ortho2align running time and peak RAM usage is quite small (Table 1) due to high degree of parallelization and dumping intermediate files into disk, which makes ortho2align feasible to run on multicore machines with restricted RAM size. Since both ortho2align and slncky use liftOver to match syntenic regions, it is unsurprising that these two tools are slower than liftOver. Resource usage of ortho2align is just slightly greater than that of slncky when predicting orthologues in mouse, which indicates no particular need in optimizing ortho2align in terms of resources.

**Table 1:** Programmes resource usage during discovery of orthologues of 14,682 human lncRNAs in different species

Programme	Subject species	Running time	Peak RAM usage
ortho2align	Macaque	02:43:05	955 Mb
ortho2align	Mouse	01:42:30	1 Gb 749 Mb
slncky	Mouse	01:31:39	1 Gb 563 Mb
liftOver	Mouse	00:00:59	1 Gb 565 Mb
ortho2align	Opossum	02:23:59	789 Mb
ortho2align	Platypus	01:19:16	534 Mb
ortho2align	Chicken	00:47:07	247 Mb
ortho2align	Xenopus	01:01:11	208 Mb

### Discovery of orthologues of novel human lncRNAs

To present practical usage and significance of our approach we aimed to predict orthologues of three distinct classes of novel human lncRNAs: X-RNAs, strRNAs and seRNAs.

**Fig. 5:**
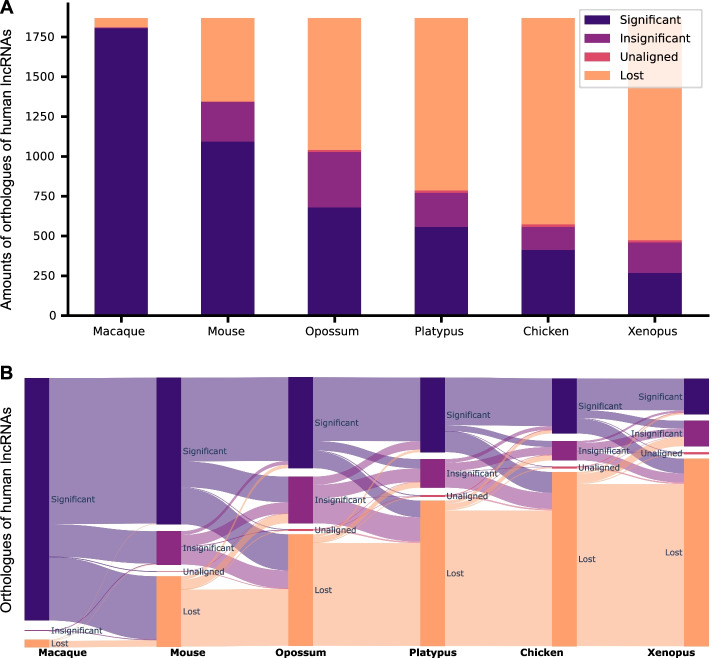
Predicted orthologues of X-RNAs in six Vertebrata species. **A** Distribution of conservation statuses across species. **B** Conservation status flow between species

X-RNAs exhibited a certain decrease in the number of significant orthologues with increase of the evolutionary distance from human to the subject species (see Fig. 5). 210 X-RNAs out of 1865 were significantly conserved in all six species and 625 X-RNAs were shown to be primate-specific. Most X-RNAs weakened their conservation statuses with increase of the evolutionary distance with a few exceptions.

In accordance with X-RNAs being defined as intergenic transcripts in a strand-specific manner, most of their orthologues were also intergenic with no regard to species or conservation status (Additional file 1: Fig. S2).

strRNAs were found to be much less conserved with no significantly conserved RNAs across all species and 34 out of 96 RNAs being primate-specific (Additional file 1: Fig. S3). Notably, PNCTR, one of strRNAs and the architectural RNA of the perinucleolar compartment, was found to be significantly conserved in all but two species, Opossum and Platypus, where it was insignificantly conserved, but successfully aligned to the respective syntenic regions.

Concerning three novel seRNAs, only one of them, peri-NPIP RNA, was significantly conserved in all six species (Additional file 1: Fig. S4). PZP antisense RNA was found to be significantly conserved in all species but Mouse, where it was insignificantly conserved. SVA D RNA has shown a stable decrease in its conservation with a total loss in Platypus and Chicken (probably due to being located at X chromosome in Human), yet unexpectedly was syntenic to a certain region in the *X. tropicalis* genome.

Annotations of lncRNAs orthologues are available in Additional file 2.

## Discussion

We developed ortho2align—a synteny-based approach for finding orthologues of novel lncRNAs with a statistical assessment of sequence conservation. Implemented strategies of restricting the search to syntenic regions, statistical filtering of HSPs and selection of orthologues provide high levels of sensitivity and specificity as well as optimal computational time even when looking for orthologues in distant species. ortho2align has shown a little bit lower TPR compared to two similar tools, which is probably due to either incorrect paralogue resolution in the benchmarking dataset or more stringent statistical HSP filtering in ortho2align pipeline compared to slncky: the latter does not apply a multiple testing correction procedure during filtering, which is statistically inaccurate. After all, the performance comparison doesn’t indicate inferiority of ortho2align to slncky or liftOver, since ortho2align is a unique tool designed for the discovery of unannotated orthologues of novel lncRNAs in distant species with no lncRNA annotation or congruent experimental data available for them, while liftOver is too naive to provide a researcher with essential information on the conservation of given lncRNAs and slncky only matches existing lncRNAs in both species and fails to predict unannotated orthologues. So it is incorrect to compare ortho2align, slncky and liftOver directly as these tools solve different problems. However, ortho2align allows for optional annotation of orthologues, so it can match existing lncRNAs in both species similar to slncky but it also provides previously unannotated orthologues unlike slncky. So it was practical to compare how ortho2align and slncky perform in a single task of matching previously annotated orthologous lncRNAs in order to assess how well ortho2align captures known orthology relationships. Since ortho2align and slncky exhibit close TPR values, we believe ortho2align is a sensitive tool for the discovery of orthologues of lncRNAs.

ortho2align is in fact a versatile tool applicable to any genomic regions, especially weakly conserved ones, not just lncRNAs. Researchers are also provided with full information about loci conservation status: either there is only syntenic relationship, two loci are alignable or their alignments are statistically significant; ortho2align also retains information about putative paralogues along with the best one-to-one orthologues selected out of them. These features render ortho2align superior to slncky and liftOver in terms of output richfullness. Versatility, small amount of input files and the completeness of information complemented with optional annotation of orthologues will allow researchers to adapt ortho2align in their orthology studies. ortho2align can also be used in bundle with other steps that researchers will consider sensible, such as the BRH strategy [19] or removal of possible protein-coding orthologues [9].

To display the predictive power of ortho2align, we predicted orthologues of three distinctive classes of novel human lncRNAs. Multiple X-RNAs were found to be conserved even in *X. tropicalis*, and their orthologues were mostly intergenic as X-RNAs themselves, which in total hints functional significance of those novel chromatin-associated RNAs. Annotation of murine X-RNAs in the RADICL [20] or GRID [21] data will allow for direct inference of human-to-mouse orthologous X-RNAs and support the supposition of their functional significance. The high degree of conservation of PNCTR, one of the most prominent strRNAs, indicates its significant role in maintaining the perinucleolar compartment in mammalian cells. Conservation of other strRNAs also suggests their putative significance in binding multiple molecules of the same protein. Finally, the analysis of conserved seRNAs might lead to the discovery of novel nuclear bodies with those seRNAs as their architectural components. Application of ortho2align to other classes of novel lncRNAs will likely be as fruitful.

## Conclusions

We developed ortho2align—a synteny-based approach for finding orthologues of novel lncRNAs of query species with a statistical assessment of sequence conservation while there is no lncRNAs annotation or congruent experimental data for subject species. The approach is sensitive and specific considering its ability to discover unannotated orthologues, which is a unique feature among similar tools. These features allowed us to predict orthologues for three distinct classes of novel human lncRNAs. The versatility and low resource usage make ortho2align suitable for a wide range of applications in orthology studies. ortho2align source code, list of dependencies, default parameter values, installation, testing and running instructions are publically available at https://github.com/dmitrymyl/ortho2align under GPL-3.0 license. ortho2align is available as Anaconda package for ease of installation and dependencies management.

## Availability and requirements

Project name: ortho2align.

Project home page: https://github.com/dmitrymyl/ortho2align

Operating system(s): Linux.

Programming language: Python.

Other requirements: Python 3.7–3.9, liftOver 377, BLAST + 2.9.0; python packages: numpy, pebble, scipy, tqdm, sortedcontainers 2.1.0

License: GNU GPL version 3.

Any restrictions to use by non-academics: None.

## Materials and methods

### Dataset of orthologues

We used a publicly available dataset of lncRNA orthologues in various species [18]. We took lncRNAs from the Supplementary dataset 1 of the aforementioned publication. We chose lncRNAs annotations of the main dataset for 7 species: human, rhesus macaque, mouse, opossum, platypus, chicken and *Xenopus tropicalis*. Due to liftOver files are being readily available only for UCSC assemblies and dataset lncRNAs were annotated in accordance with Ensembl assemblies, we mapped Ensembl genome versions to UCSC genome versions (Additional file 1: Table S1) and manually converted chromosome names from Ensembl to UCSC nomenclature with some lncRNAs in contigs being lost (Additional file 1: Table S2). We then prepared tables mapping names of orthologous lncRNAs in human and every other species from one-to-one lncRNA families data. Final annotation and mapping data is available in Additional file 3.

To produce species tree in Figs. 2 and 3, we downloaded species tree from Ensembl Compara release 106 and manually removed unnecessary species branches with MEGA X [22].

### Quality metrics definition

A true positive hit is defined as a predicted orthologue intersecting a correct orthologue in a strand-specific manner. A false positive hit is defined as an orthologue predicted for a lncRNA with no annotated orthologues in the dataset. TP is defined as a number of true positive hits, FP is defined as a number of false positive hits, TN is defined as a total number of non-orthologous lncRNAs (total_non_ortho) minus FP and FN is defined as a total number of orthologous lncRNAs (total_ortho) minus TP. Ratio metrics are defined as follows: TPR = TP / total_ortho, FPR = FP / total_non_ortho, TNR = TN / total_non_ortho, FNR = FN / total_ortho, accuracy = (TP + TN) / (TP + TN + FP + FN), precision = TP / (TP + FP), F1 = TP / (TP + 0.5 * (FP + FN)).

Since liftOver by default discards duplicated genomic ranges, that might come from genomic duplications and result in paralogues, it was decided to allow liftOver to retain all duplicated genomic ranges after lifting. So, liftOver might find multiple orthologues for a single lncRNA, so its TP and FP values are weighted after calculating TN and FN. Every lncRNA contributes not a “1” to TP or FP, but a share of a true (or false) orthologue(-s) to the found orthologues.

### Benchmarking settings

ortho2align v1.0.4 was run with run_pipeline subcommand. Default parameter values were used except for min_ratio, pval_threshold, and FDR correction. The best min_ratio value was selected between 0.01 and 0.05. The best pval_threshold value was selected among the following values: 1e-6, 1e-2, 5e-2, 1e-1, 2e-1. Selection of the best values of those two parameters was conducted jointly by maximizing TPR for each species separately (see Additional file 1: Table S3 for the final values). FDR correction was enabled. Background genomic ranges were constructed from the subject species lncRNAs annotation files from the benchmarking dataset. The same annotation files were used for the annotation of found orthologues. The programme was parallelized on 20 cores. Running time and RAM consumption were recorded by the programme itself.

slncky v1.0.4 was run with key -2 (no filtering, only searching for orthologues) and default parameter values. Annotation and genome files that were used were provided with slncky except for the annotation of noncoding RNAs in mm9, which was replaced with mm9 lncRNAs annotation from the benchmarking dataset. The programme was parallelized on 20 cores. Running time and RAM consumption was recorded with the programme “GNU time 1.7”.

liftOver v377 was run with the following parameters: -multiple -noSerial -minMatch = 0.05 (a default minMatch value in the UCSC online utility). Enabling flag “-multiple” allowed retention of duplicated genomic ranges, possible paralogues. Predicted orthologues were annotated with bedtools intersect -s -loj -a < predicted_orthologues > -b < true_orthologues > , where < true_orthologues > is the mm9 lncRNAs annotation from the benchmarking dataset. Running time and RAM consumption was recorded with the programme “GNU time 1.7”.

Genome fasta files and liftOver chain files for respective genome versions were downloaded from the UCSC website and were used in ortho2align and liftOver.

### Discovery of orthologues of novel human lncRNAs

We have downloaded annotations of X-RNAs (1865 pcs), strRNAs (96 pcs) and seRNAs (3 pcs) from the respective publications. We aimed at predicting their orthologues in the most recent genomes assemblies of species (Additional file 1: Table S4), yet corresponding liftOver chain files were available only for hg38 assembly. As X-RNAs and seRNAs were annotated for hg19 assembly, we converted them to hg38 assembly with liftOver (only two out of 1867 X-RNAs and no seRNAs were lost). strRNAs were already annotated for hg38 assembly. We ran ortho2align with parameters selected during the benchmarking process. To annotate predicted orthologues we used RefSeq annotations for the corresponding genomes (Additional file 1: Table S4) with chromosome names converted between RefSeq and UCSC systems using UCSC chromAlias files and a custom script. Only genes (i.e. records with gbkey = “Gene”) were used for the annotation process.

## Supplementary Information

**Additional file 1**. **Fig. S1.** ortho2align pipeline composition. **Fig. S2.** Annotation status of X-RNAs orthologues across species and conservation statuses. **Fig. S3.** Predicted orthologues of strRNAs in six Vertebrata species. **A.** Distribution of conservation statuses across species. **B.** Conservation status flow between species. **Fig. S4.** Unannotated seRNAs orthologues’ conservation statuses. **Table S1.** Genome versions used in benchmarking. **Table S2.** lncRNAs orthologues dataset statistics. **Table S3.** Best parameter values of ortho2align in terms of TPR maximization. **Table S4.** Genome versions used in predicting orthologues for novel human lncRNAs.

**Additional file 2**. This ZIP archive contains output files of ortho2align search for orthologues of three novel classes of lncRNAs: X-RNAs, strRNAs and seRNAs – in six species listed in the main text including genomic coordinates of those orthologues.

**Additional file 3**. This ZIP archive contains files used for benchmarking – annotations of lncRNAs in six species in BED format along with TSV tables of orthologous correspondence between human lncRNAs and those of other species.

## Data Availability

All data generated or analysed during this study are included in this published article and its supplementary information files.

## Abbreviations

HSP: High-scoring segment pair
TP: True positive
FP: False positive
TN: True negative
FN: False negative
TPR: True positive rate
FPR: False positive rate
FNR: False negative rate
TNR: True negative rate
Acc: Accuracy
Pre: Precision
F1: F1-score
X-RNAs: Novel chromatin-associated RNAs
strRNAs: Short tandem repeat-enriched RNAs
seRNAs: Semi-extractable RNAs
