# Supplementary material for: ortho2align: a sensitive approach for searching for orthologues of novel lncRNAs
Source: BMC Bioinformatics. 2022 Sep 19;23:384. doi: 10.1186/s12859-022-04929-y (PMC9487038; doi:10.1186/s12859-022-04929-y)
Supplement: Supplementary file 1 — Additional file 1. Fig. S1. ortho2align pipeline composition. Fig. S2. Annotation status of X-RNAs orthologues across species and conservation statuses. Fig. S3. Predicted orthologues of strRNAs in six Vertebrata species. A. Distribution of conservation statuses across species. B. Conservation status flow between species. Fig. S4. Unannotated seRNAs orthologues’ conservation statuses. Table S1. Genome versions used in benchmarking. Table S2. lncRNAs orthologues dataset statistics. Table S3. Best parameter values of ortho2align in terms of TPR maximization. Table S4. Genome versions used in predicting orthologues for novel human lncRNAs. [file 12859_2022_4929_MOESM1_ESM.pdf]

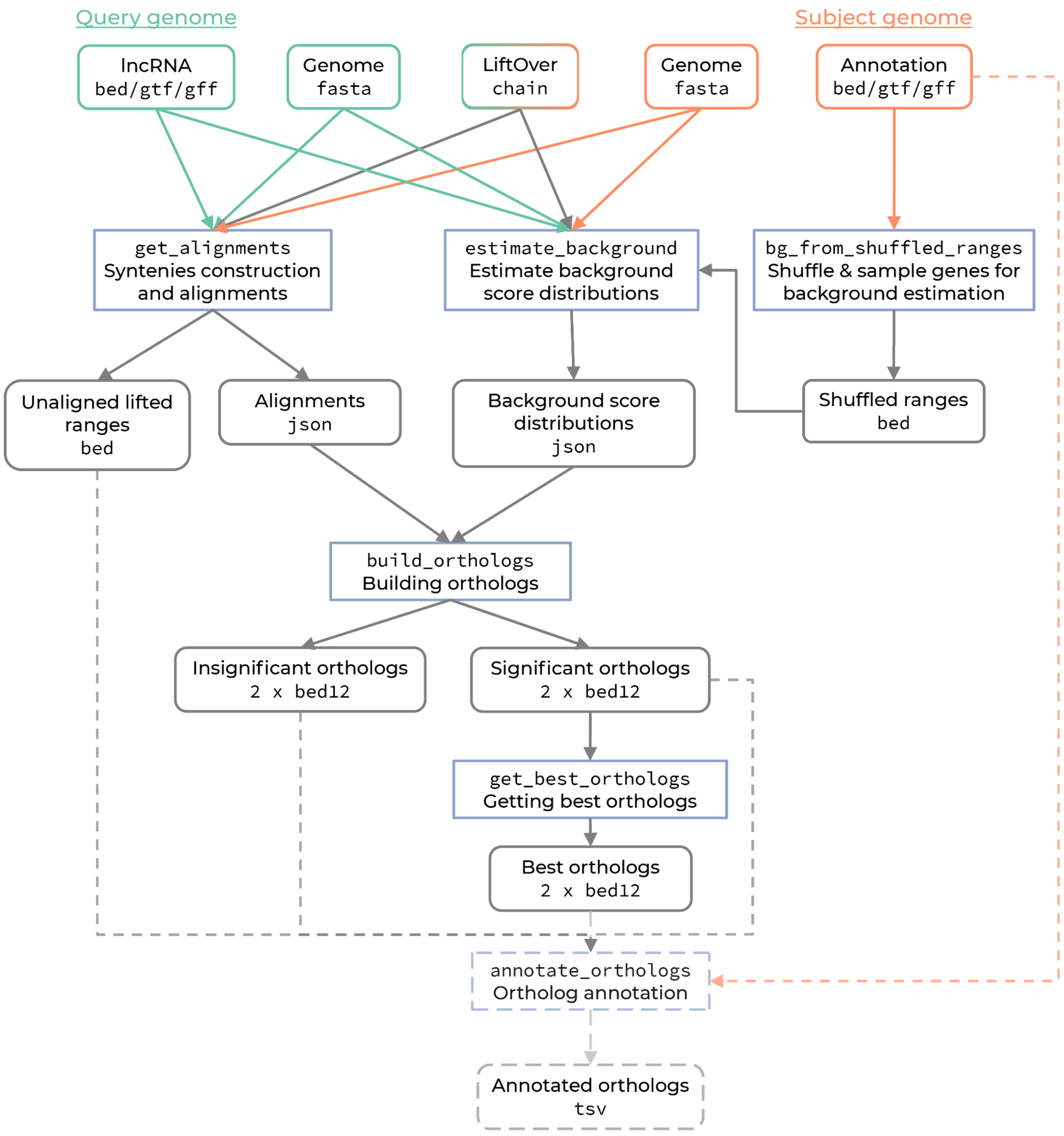

Figure S1. ortho2align pipeline composition.

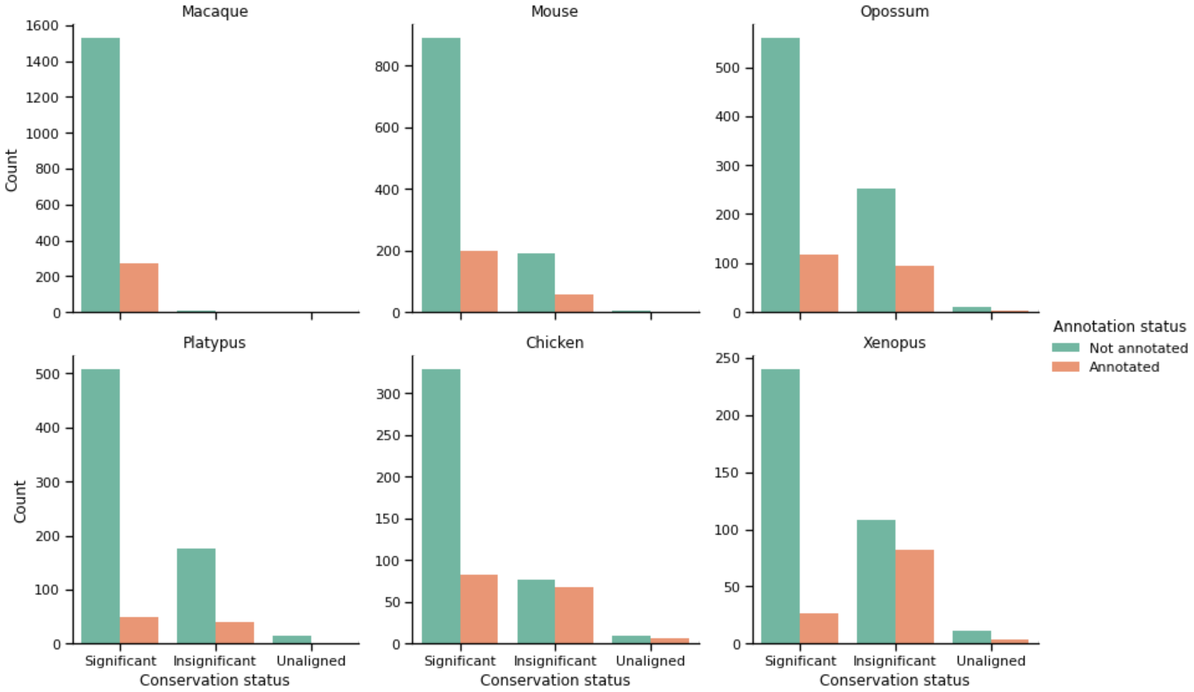

Figure S2. Annotation status of X-RNAs orthologues across species and conservation statuses.

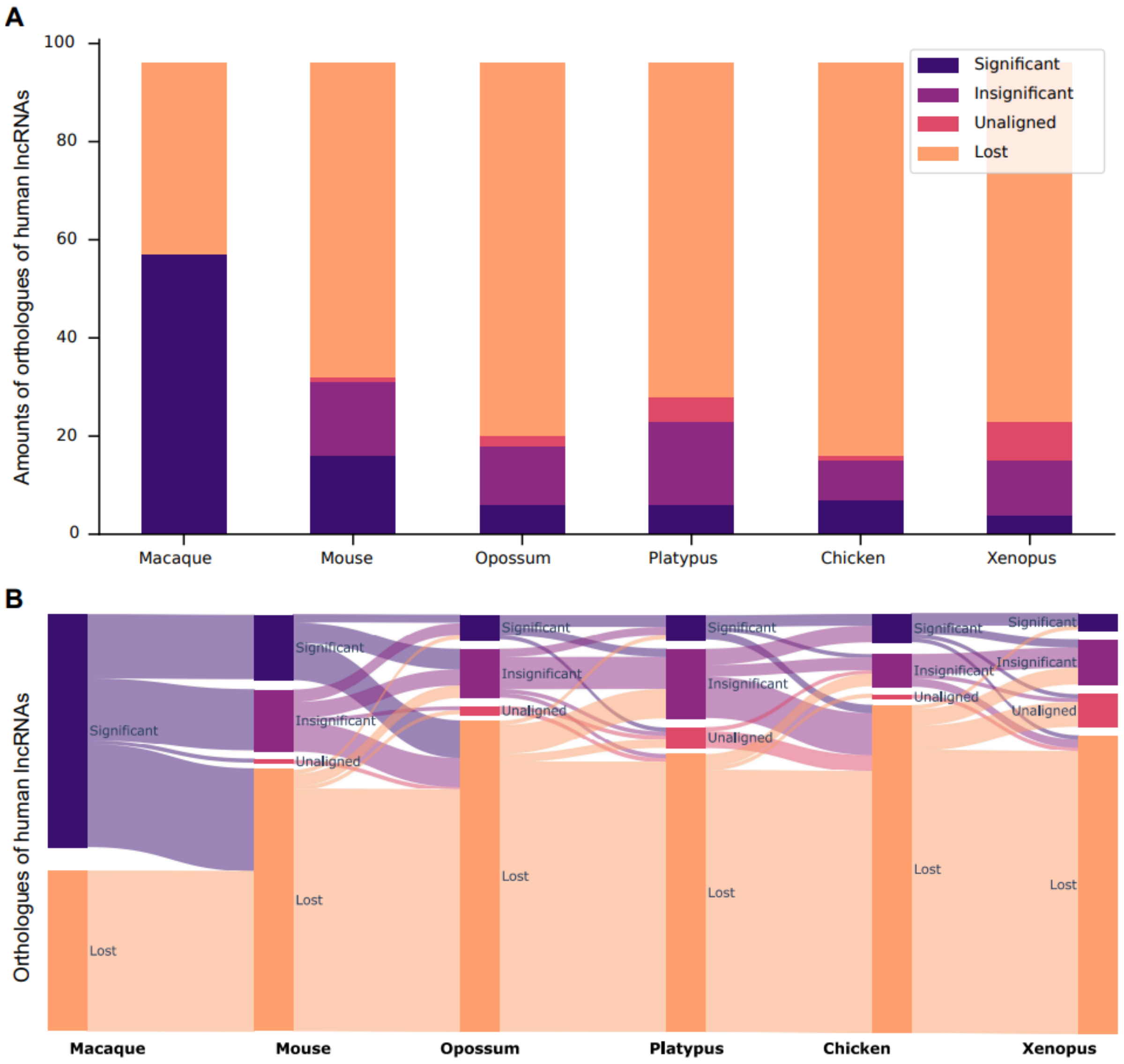

Figure S3. Predicted orthologues of strRNAs in six Vertebrata species.
A. Distribution of conservation statuses across species. **B.** Conservation status flow between species.

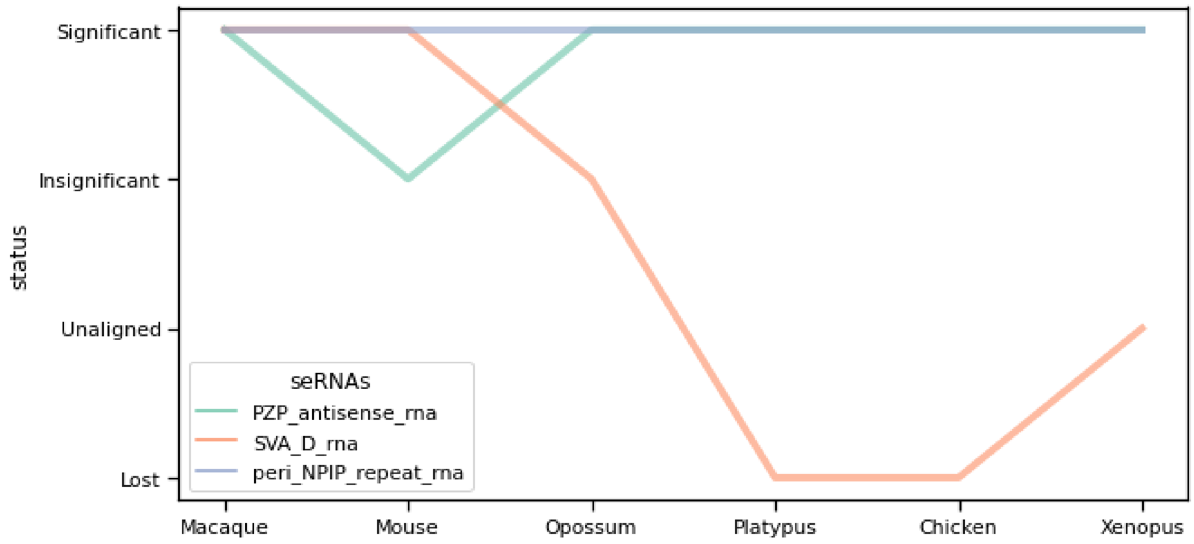

Figure S4. Unannotated seRNAs orthologues' conservation statuses.

Table S1. Genome versions used in benchmarking.

Species	Ensembl genome version	UCSC genome version	Assembly level
Human	GRCh37	hg19	Chromosome
Macaque	MMUL 1.0	rheMac2	Chromosome
Mouse	NCBI37	mm9	Chromosome
Opossum	monDom5	monDom5	Chromosome
Platypus	OANA5	ornAna1	Chromosome (78% in scaffolds)
Chicken	WASHUC2	galGal3	Chromosome
Xenopus	JGI 4.2	xenTro3	Scaffold

Table S2. lncRNAs orthologues dataset statistics.

Species	lncRNAs, total	lncRNAs lost in chromosome names conversion	lncRNAs retained in chromosome names conversion	Orthologues of human lncRNAs
Human	14682	0	14682	Not applicable
Macaque	15280	559 (from contigs)	14721	12868
Mouse	10850	9	10841	2720
Opossum	8039	0	8039	1261
Platypus	6889	32 (from contigs)	6857	823
Chicken	5412	0	5412	580
Xenopus	3296	0	3296	204

Table S3. Best parameter values of ortho2align in terms of TPR maximization.

Species	min_ratio	pval_threshold
Macaque	0.05	0.2
Mouse	0.01	0.2
Opossum	0.01	0.1
Platypus	0.01	0.05
Chicken	0.01	0.1
Xenopus	0.01	1e-6

Table S4. Genome versions used in predicting orthologues for novel human lncRNAs.

Species	UCSC version	RefSeq version
Macaque	rheMac10	GCF_003339765.1
Mouse	mm10	GCF_000001635.26
Opossum	monDom5	GCF_000002295.2
Platypus	ornAna2	GCF_000002275.2
Chicken	galGal6	GCF_000002315.5
Xenopus	xenTro10	GCF_000004195.4
